# The Human Self Has Two Serial Aspects and Is Dynamic: A Concept Based on Neurophysiological Evidence Supporting a Multiple Aspects Self Theory (MAST)

**DOI:** 10.3390/life11070611

**Published:** 2021-06-24

**Authors:** Peter Walla, Georg Northoff, Cornelia Herbert

**Affiliations:** 1 Faculty of Psychology, Sigmund Freud University Vienna, Freudplatz 1, 1020 Vienna, Austria; 2 Faculty of Medicine, Sigmund Freud University Vienna, Freudplatz 3, 1020 Vienna, Austria; 3 Centre for Translational Neuroscience and Mental Health Research, School of Psychology, University of Newcastle, Callaghan, NSW 2308, Australia; 4 Mind, Brain Imaging and Neuroethics, Institute of Mental Health Research, University of Ottawa, Ottawa, ON K1H 8M5, Canada; georg.northoff@theroyal.ca; 5 Department of Applied Emotion and Motivation Psychology, Institute of Psychology and Education, Ulm University, 89081 Ulm, Germany; cornelia.herbert@uni-ulm.de

**Keywords:** self, brain imaging, EEG, multiple aspects, self-referential processing, neuroscience, neurobiology, Me1 and Me2

## Abstract

The self is an increasingly central topic in current neuroscience. Understanding the neural processes that are involved in self-referential processing and functioning may also be crucial to understanding consciousness. The current short communication goes beyond the typical concept that the self is singular, as has been assumed from neuroanatomical descriptions of the self by fMRI and PET studies. Long ago, theoretically, the idea of multiple aspects of the human self-arose, highlighting a dynamic organizational structure, but an increasing number of electrophysiological brain imaging studies, searching for the temporal dynamics of self-referential brain processes, now has empirical evidence supporting their existence. This short communication focuses on the theoretical idea of a dynamic self and provides first preliminary empirical evidence, including results from own studies of the authors, in support of, and highlights the serial dynamics of the human self, suggesting a primitive Me1 and an elaborate Me2 (a non-personal and a personal self). By focusing on the temporal dimension of the self, we propose that multiple aspects of the self can be distinguished based on their temporal sequence. A multiple aspects Self Theory (MAST) is proposed. This model is meant as a theoretical framework for future studies providing further support.

## 1. Introduction

Without a doubt, previous brain imaging research has completed existing philosophical and psychological concepts of the singular human self by demonstrating consistent underlying functional neuroanatomy. Numerous studies were conducted by utilising functional magnetic resonance imaging (fMRI) and positron emission tomography (PET), e.g., [1,2,3,4,5]. These methods are well recognised for their excellent spatial resolution, but at the same time, they are also known for their poor temporal resolution, which might explain why no study based on these methods has provided evidence that a series of chronological processes underlies the elaborate sense of self. Therefore, multiple aspect theories about the human self that were formulated more than 100 years ago by remarkably influential people, such as William James [6] and Sigmund Freud [7], have laid dormant in the neurosciences for over a century.

How is the self really related to the brain? Beyond the spatial dimension, one may want to tap into the temporal dimension of neural activity in order to support any multiple aspect theory of the human self. The human mind has a hierarchical, functional structure, and depends on serial processing taking place on different levels at different locations in the brain. Furthermore, the temporal range of neural processing in the brain is certainly more within the millisecond—than the multiple second—range. Thus, only brain imaging with high temporal resolution is able to differentiate temporally separate brain activities. This becomes particularly crucial for understanding how complex brain functions (such as self-referential processing) are, which likely involves multi-level brain activities, some of which happen in series, while others occur at almost the same time, being separated by only fractions of a second.

Despite the valuable description of neuroanatomical structures that are involved in self-referential processing, as found via fMRI and PET studies, (see [2]), most of the findings can only be seen as supporting a merely single-aspect concept of the human self when the temporal aspects of self-processing are considered. However, the absence of evidence is not an evidence for the absence of such multiple and temporally serially organised self-aspects. Even though William James’ scientific toolbox did not contain any objective neurophysiological methodology, it seems his original theory that the self involves an objective “Me” and a subjective “I” (as mirrored in the distinction between subjective self and objective self (see [8]) has, in fact, found support via recent electrophysiological investigations. The current short communication paper focuses on the temporal aspect of self-processing, as examined in studies using electroencephalography (EEG) and magnetoencephalography (MEG), and suggests a theoretical framework that describes the dynamic dimension of self-referential processing. As highlighted in this short communication, many results from previous electrophysiological research support the idea that there are multiple aspects of the human self. In line with this evidence, we highlight research from our own empirical findings, which leads us to suggest a multiple aspects self-theory (MAST). The MAST is based on electrophysiological findings, including our own findings, and therefore is meant to form a basis and guide for future studies investigating the dynamics underlying self-referential processing.

## 2. Brain Imaging and the Transition from a Single Aspect Self Concept to the Dynamic Self

**Figure 1:**
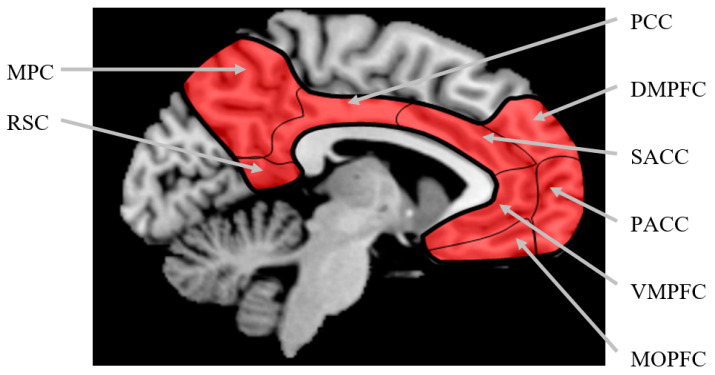
Cortical midline structures marked in red colour. MOPFC = medial orbital prefrontal cortex, VMPFC = ventromedial prefrontal cortex, PACC = pre- and subgenual anterior cingulate cortex, SACC = supragenual anterior cingulate cortex, DMPFC = dorsomedial prefrontal cortex, MPC = medial parietal cortex, PCC = posterior cingulate cortex, RSC = retrosplenial cortex. Note that there are no clear anatomically defined borders between the different regions. Adapted from Northoff et al. [2].

Since the beginning of the third millennium, a number of brain imaging studies using fMRI and PET were published about brain regions associated with the human self. In 2001, for instance, Vogeley et al. [9] explored distinct and overlapping brain regions associated with theory of mind and self-perspective. Kelley et al. [10] scanned their participants (n = 21) while they made judgements about trait adjectives that either had self-relevance, other-relevance, or were simple case judgements. Any relevance judgements were associated with increased left inferior frontal cortex activity in addition to increased anterior cingulate activity when compared to case judgements. A self-referential specific activity could be identified at the medial prefrontal cortex. Both of these studies induced self-reference via language tools. Others used various different approaches, such as the rubber hand illusion [11], movie clips [12], imagination of action [13], self-related emotion [14], and liking of food [15] to elicit self-referential processing. Northoff et al. [2] ran a meta-analysis on 27 of these studies (7 PET and 20 fMRI) published between 2001 and 2005 and found common activation in cortical midline structures across all studies that occurred in association with self-referential processing, regardless of stimulus modality and task domain. These cortical midline structures include the medial orbital prefrontal cortex, the ventromedial prefrontal cortex, the pre- and subgenual anterior cingulate cortex, the supragenual anterior cingulate cortex, the dorsomedial prefrontal cortex, the medial parietal cortex, the posterior cingulate cortex, and the retrosplenial cortex (see Figure 1).

A larger network of brain regions, including these cortical midline structures, in addition to the tempo-parietal junction and the anterior temporal gyrus, has been defined as the so-called social brain. This network was found to be more active during processing of social words versus non-social words [16]. More specifically, the processing of words that reflected each participant’s individual group belonging (e.g., religious group or nationality) versus out-group words was associated with activity at the ventral medial prefrontal and the anterior and dorsal cingulate cortex. These structures are again part of the cortical midline network, and the respective findings thus confirm their engagement in self-referential processing while even extending it to a social context. Interestingly, Raichle et al. [17] introduced the science community to the so-called default mode network, which showed consistent deactivation across brain structures, such as the posterior cingulate cortex, the medial prefrontal cortex, and the medial, lateral, and inferior parietal cortex, while one is not focussed on the outside world and not engaged in any task (see also [5,18,19,20]). These areas seem to have a high degree of functional connectivity in the brain’s resting state. The striking thing is that the common deactivation in this neural network turns into activity during self-referential processing (e.g., [21]) as in first-person perspective [22] and sense of agency [23].

In summary, a number of studies provide valuable and remarkably robust insight into self-referential processing in the human brain with high significance, but as mentioned above, none of them revealed anything that points to a multiple aspect existence of the human self. Perhaps the reason for this lies in the nature of the brain imaging methods that were utilised, and partly also in the nature of the experimental paradigms. If the human self indeed has more than just one aspect, then one would expect these multiple aspects to be tightly linked and temporally separated by only fractions of a second. As already mentioned, both fMRI and PET lack respective temporal sensitivity to detect subtle functional differences between possible multiple self-aspects. Other alternative methods must be utilised to search for these. The only alternatives in terms of measuring neural activity are MEG and EEG. These methods are well known for their excellent temporal resolution and are both well suited to test whether the human self indeed has more than just one aspect. Only some years ago have researchers begun to utilise these neurophysiological brain imaging technologies that directly measure brain activity in contrast to increased blood flow as a consequence of neural activity, which is what fMRI and PET are sensitive to, in order to gain further insight into the dynamics of the human self. Only with neurophysiological imaging can one describe the spatiotemporal features underlying self-referential processing.

As has been summarised by Knyazev [24], some of the EEG work that has been done to investigate self-referential processing strongly supports the idea of cortical midline structures to be engaged in self-referential processing, which supports and confirms the above-mentioned cortical midline structure network. His review emphasises that EEG correlates of self-referential processing were most commonly found in the medial prefrontal cortex. He further mentions that the well-known P300 ERP component has been mostly associated with differences between self and other. Some authors report about rhythmic brain activity related to self-referential processing (e.g., [25,26]). As pointed out by Knyazev [24] in his summary of EEG studies, there is substantial evidence about self-referential processing. However, still, a theoretical framework describing the dynamic and multiple aspect nature of the self is missing and seems helpful. This is exactly what the present short communication paper concentrates on.

## 3. The Neurophysiological Concept of the Multiple Aspect Theory of the Self

Since 2007, a number of studies have indeed provided empirical evidence about the existence of a multiple aspect self [27,28,29,30,31,32,33,34,35]. Interestingly, all of those studies used possessive pronouns to elicit self- versus other-referential processing. Possessive pronouns mean ownership and thus allow to easily induce neural processing reflecting self (my), other (your), or nobody (a). Using possessive pronouns as stimuli and similar experimental paradigms resulted in the distinction of two aspects of the self (for details see Section 4 below). In addition, there is even evidence that highlights chronological aspects of self-referential processing, or in other words, the serial dynamic nature of the self. Thereby, it is suggested that one self-aspect is activated before the other, and in case of parallel processing, one is more basic than the other, which also leads to an evolutionary view that one is more primitive than the other. This has potential implications for various developmental and clinical fields, because a serial dynamic self-concept means that a disordered self, as characterised by many psychiatric conditions, may be associated with deficits of only one self-aspect (the earlier or the later/the more primitive or the more elaborate) or abnormalities related to both aspects of self. A distinction between two serial self-aspects could be essential to fully understand and provide targeted treatment for such disorders. In addition, this could provide greater insight into early human development and potential self-referential processing in non-human animals and thus could lead to a very different perception of non-human animal capacities in principle. The following section elaborates on the spatiotemporal properties of the human self, introduces the concept of a serial dynamic self of a primitive Me1 and an elaborate Me2, and proposes a multiple aspects self-theory (MAST). This MAST is understood as a theoretical concept about self-referential processing in the human brain, including all above—and below—mentioned features as a result of existing literature on the serial and dynamic electrophysiological findings related to self-referential processing.

## 4. Spatiotemporal EEG and MEG Activity Patterns Providing Empirical Evidence to Support the Idea of a Serial Dynamic Self

As mentioned earlier, William James introduced the concept of distinguishing between “I” and “Me” (1890) [6]. In other words, he believed that the self is rather a complex composition of at least two separate aspects than a single entity, or that two different kinds of self exist. The high temporal resolution of both EEG and MEG allowed researchers to provide promising electrophysiological evidence that finally gives reason to believe that the earliest ideas about how the self in the human brain is mediated (or constructed) may prove right after all. However, it is not yet clear whether this first empirical evidence can be interpreted in terms of aspects of one kind of self or it should rather be seen as different stages of self, one more primitive than the other. The latter view would nicely fit into an evolution-based concept of the self that supports the idea of developmental self-stages, and seems more likely given the existing evidence.

As already mentioned, some of the fMRI and PET studies used language tools to induce self-referential processing, but most often, their experimental paradigms included rather complex stories. In contrast, first specific support for the theory of a serial dynamic self comes from electrophysiological studies that induced self- or other-related processing through ownership or belonging, by visually presenting possessive pronouns and nouns (only word pairs). Reading “a car” leaves one in the dark regarding who that car belongs to, and thus neither “other” nor “self” referential processing is induced. On the other hand, “his car” clearly indicates the engagement of another person, whoever that may be. Crucially, reading “my car” elicits self- referential processing. This approach provides a simplified induction of self-referential processing, and in this case, simple may be better, because complex scenarios elicit a range of other brain activities that can distort or bias pure self-referential processing. Walla et al. translated the simple ownership idea into an experimental paradigm and first ran a MEG study [27], soon after followed by an independent EEG study [28] with a different participant population. Strikingly, significant spatiotemporal neurophysiological differences were found first between no personal reference (“a car”) and any personal reference (“his car” and “my car”), and second between “other” reference and “self” reference (“his car” different from “my car”). Furthermore, these two findings emerged in brain activity at different times. The first brain activity difference between no and any personal reference occurred over occipitoparietal brain areas and at an early post stimulus delay (about 200 to 400 ms after word onset). The brain activity difference between “his” versus “my” was found over the left frontotemporal area at around 600 ms after word onset. Both electrophysiological methods produced highly similar findings and because EEG and MEG methods provide equally high temporal resolutions, the second study (EEG) is considered a reliable replication of the first study (MEG).

**Figure 2:**
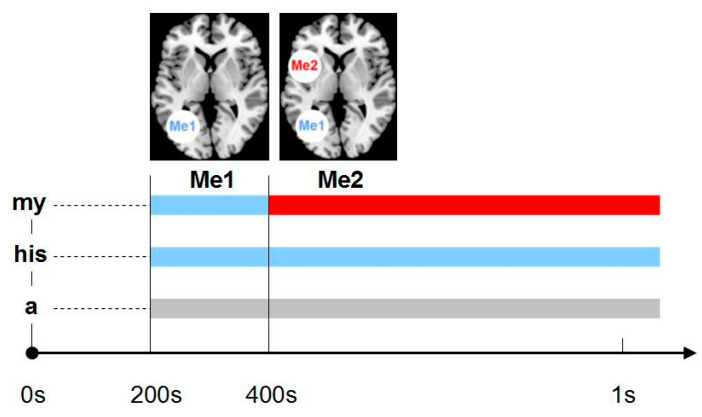
Schematic model depicting Me1and Me2 locations and temporal features.

The early stage of any personal engagement is here referred to as the Me1, whereas the later stage reflecting an elaborate sense of self is referred to as the Me2 (Figure 2). Walla et al. [27,28] suggested that the neuroanatomical substrate for Me2 processing could be the insular cortex. The insular cortex has been described as being engaged in interoceptive awareness of body states (e.g., [36]) and, thus, seems to be a likely candidate for participating in self-referential processing. Karnath et al. [37] reported about the insular cortex mediating awareness of movements of one’s own limbs. They already suggested that this structure might be integral to self-referential processing. A year earlier, Singer et al. [38] found parts of the insular cortex to be involved in a network of empathy-related processing, such as observing others experiencing pain. It is thought that the insular cortex within empathy-related processing mediates the link between the external and internal world, which gives it another aspect in terms of self-referential processing, in particular, it connects the self with emotion.

**Figure 3:**
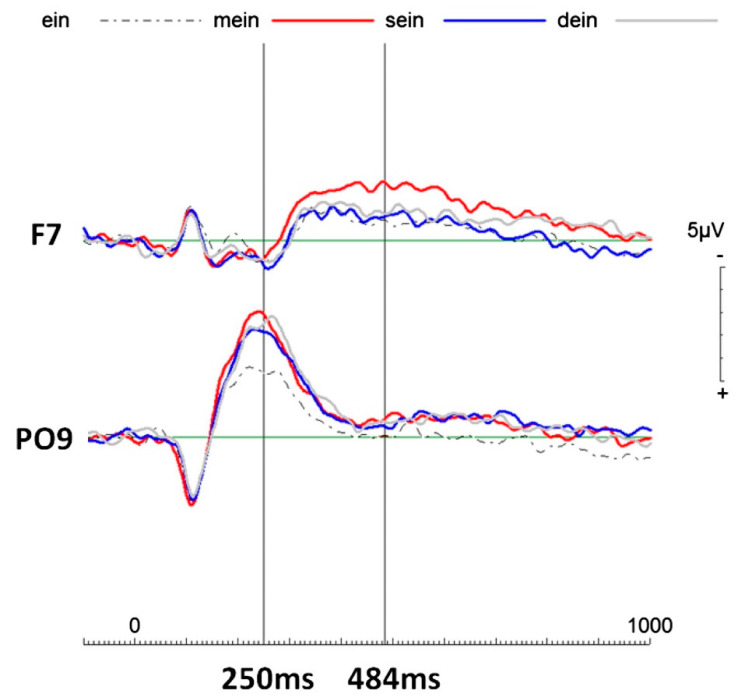
Event-related potentials (ERPs) of all four pronouns of interest (possessive pronouns: “mein” (my), “sein” (his), “dein” (your), and non-personal pronoun “ein” (a)). Notes: around 250 ms post-stimulus onset all possessive pronouns elicited more negative going electrophysiological activity compared to the non-personal pronoun “ein” (a) over the left parieto-occipital area (i.e., PO9 electrode location). This finding is here referred to as Me1, which simply reflects any personal engagement. Later, starting at about 350 ms post-stimulus, “mein” elicited more negative going activity than the rest over the left frontal cortical area. This finding is here referred to as Me2, the proper (elaborate) self. Figure taken from Walla and Herbert [48], shared first authorship and correspondence, supported by the German Research Foundation (DFG HE5880/3-1) and the Gustav-A. Lienert-Stiftung awarded to Herbert, C.

In 2008, Esslen et al. [29] reported in their study about pre-reflective and reflective self-aspects that the pre-reflective self, which they also call the minimal self, might be present in lower species (as they might experience some degree of existence), whereas what they call the reflective self can only exist in at least partially self-conscious species. Such a neurobiological perspective of self-referential processing is well supported by the Walla et al. findings [27,28]. In both studies, evidence was shown that the Me1 was actually no different to other in contrast to the Me2, which differed from other. It has been concluded that the Me1 may simply reflect biological existence or “being” a living creature instead of being an object, while the Me2 forms the neurophysiological basis for a proper subjective self (or elaborate sense of self) (see Figure 3). While it may seem early at this stage to form a solid hypothesis about a serial dynamic self, it is important to mention that these findings are strongly supported by a number of subsequent studies that are summarised in the following.

First, Zhou et al. [33] provided further EEG evidence demonstrating brain activity differences based on different possessive pronouns being visually presented to their study participants. Grand-averaged brain potentials referenced to a common average demonstrated a larger P300 component in response to passive reading of the Chinese word “wo de” (Chinese for “my”/“mine”) compared to the Chinese word “ta de” (Chinese for “his”). The authors used an oddball paradigm, in which the possessive pronouns were the rare stimuli.

Another study by the same group [34] demonstrated again that brain processes elicited by “wo de” are different from brain processes elicited by “ta de”. This time, simple differences between the characters that comprise these two words were ruled out to demonstrate more clearly that the semantic content caused differences in brain activities rather than pure character differences. In their abstract, the auth35ors report about activities in medial prefrontal, anterior cingulate and post-central cortex. Their actual figure though in the manuscript depicting low-resolution electromagnetic tomography (LORETA) solutions for activity differences between self- and other-related pronouns shows various other regions of temporal and frontal lobe structures to be engaged in self-referential processing. Unfortunately, in both studies, no neutral condition was included and thus it could not be tested whether or not both possessive pronoun conditions differ similarly from a neutral (non-possessive) condition to support the idea of the Me1 at an early processing stage. However, they showed strong support for the existence of the Me2 and that even possessive pronouns alone (without associated nouns) elicit brain processes that discriminate between self and other when measured with EEG. 

Yet, other EEG-LORETA studies using German pronouns of the first, second, or third person to investigate self-referential and other-referential pronoun-processing in the visual as well as in the auditory modality under both, spontaneous and controlled processing [39,40], support early and late self-referential and other-referential processing differences that in part could be modality-specific. In line with early and late stages of self-referential processing, EEG-LORETA analysis confirmed activity changes in the CMS to be involved in self-referential processing as well as in self-other-referential processing when processing personal and possessive pronouns of the first- vs. second vs. third person [40]. 

An earlier study about self-relevant object recognition provided evidence for a distinction between an early more primitive Me1 and a later more elaborate Me2 [34]. The researchers presented their participants with objects that were either their own, or familiar, but not their own, or very unfamiliar. The pattern of their ERP findings strongly resembles that found in both Walla et al. studies [27,28]. It must be emphasised though that a direct comparison only makes sense under the assumption that the familiar object condition (not one’s own object) in Miyakoshi et al.’s study is similar to the “someone else possesses an object” (e.g., “his car”) condition in both Walla et al.’s studies, and also that the unfamiliar object condition is similar to the “no one possesses an object” condition (e.g., “a car”). In line with Walla et al.’s studies, Miyakoshi et al. [41] found an early left posterior brain activity difference (at around 200 to 300 ms post stimulus) between the familiar object conditions (familiar owned and familiar not owned) and the unfamiliar condition, and a later ERP difference (at around 300 to 700 ms post stimulus) between familiar owned and familiar not owned, or in other words between self and other.

Further support for the present model of a serial dynamic self, comprising a primitive Me1 and an elaborate Me2, comes from studies from Herbert et al. [30,31] that combined the person reference paradigm (or ownership) with emotion-related information processing. In search of the self, many imaging studies investigated self-referential processing of (and with) emotional stimuli, but only a few of these studies aimed to investigate the interaction between self- and emotion-related processes from a dynamic perspective and without using explicit self-referential instructions. Of the existing studies summarised in past and recent meta-analytic research, the majority of studies considered used self-evaluation paradigms comprising emotional adjectives, and an instruction asking participants to indicate which of the presented emotional adjectives best described their personality (for an overview see, e.g., [2]. Some used emotional pictures instead of adjectives in combination with the self-evaluation instruction to evaluate the picture content for subjective and individual relevance. In a number of other studies, participants were asked to appraise emotional stimuli from either a first or a third person perspective, often in combination with the instruction to regulate the feelings elicited during picture viewing.

The respective studies by Herbert et al. [30,31,32] investigated changes in brain activity as a function of a word’s emotional significance and its self-reference, using both EEG and imaging methodology. In each of the studies, both stimulus dimensions were experimentally manipulated in a stimulus-driven way by the use of pronoun–noun pairs. Pronoun–noun pairs could describe one’s own or another person’s feelings, such as in the examples “my fear” and “his fear”, or were of neutral valence (e.g., my picture, his picture). Pronoun–noun pairs were presented together with article–noun pairs, which contained no personal reference at all. Article–noun pairs thus varied in terms of emotional valence only, from unpleasant to pleasant to neutral, such as in the examples “the fear”, “the success”, and “the picture”. Stimuli belonging to the different emotional valence and self-other categories were matched for linguistic dimensions. There was no further instruction given to the participants than to read the words silently.

An fMRI study using this paradigm in healthy male and female participants [31] revealed amygdala and insula activity (bilaterally) during processing of unpleasant words regardless of their personal reference and relatedness (self, other, no reference) and for pleasant words only in self-related emotion conditions. Processing of self-related emotional words, (unpleasant and pleasant ones), however, selectively enhanced activity in the ventral MPFC. Ventral MPFC activation included adjacent voxels in the vmACC. Notably, activation in these ventral MPFC regions was not observed during processing of self-related neutral words, which compared to self- and other-related emotion words produced deactivation in these regions, as did neutral article-noun pairs, which were personally unrelated.

The results of this imaging study [31] using words as stimuli support a role of the amygdala as a an emotion processing brain structure [39] and the insula as a brain structure involved, amongst others, in the processing of interoceptive and bodily feelings [35] and the CMS in emotional self-reference processing, an idea that has been discussed theoretically and empirically in meta-analytic studies (e.g., [2]). The novel finding of the study [31] is that it shows that activity changes in the CMS, the ventral affective medial prefrontal network in particular, are not dependent on task-induced self-referential processing or on any kind of explicit self-referential processing instructions. Instead, the results show that the CMS is also activated spontaneously in a bottom-up like fashion during the processing of symbolic information (words), making a reference to the reader’s own emotions. As there was no instruction to appraise the meaning of the words in a certain way, but rather the instruction was to read the words silently, this is strong evidence for the concept of the CMS and its subsystems, as well as the role this brain system has been hypothesised to play in self-referential processing. Thus, CMS networks are not artificial products from meta-analytic conclusions. On the contrary, changes in activation of these brain structures occur also in the absence of any self-referential processing instructions. This raises questions about the functional relevance of the CMS system. According to some authors, activation of the vmPFC and vmACC mirrors the transition from unconscious to conscious emotion processing and from non-self to self-awareness [30,31]. It is therefore likely that activation of the vmPFC during reading of self-related emotion words such as “my fear” or “my happiness”, as compared to reading of emotion words such as “his fear” or “the fear”, indicates the process by which emotional information becomes embodied (bodily integrated) and transformed into a “feeling”, and the awareness that this feeling is distinct from others and belonging to the self [31]. Viewed from the perspective of a serial dynamic self, propagated in this paper, activation of MPFC regions during reading of self-related emotions should be related more with the ME2 and, thus, the elaborate self than with the primitive ME1.

This is also what converging electroencephalographic (EEG) studies e.g., [30] found. Using the same paradigm as described above, these studies determined the processing stages of emotion x self-interactions and the temporal dynamics of this interaction’s underlying processes. Using neutral nouns related to self, other, or nobody, as in the studies by Walla et al. [27,28], in addition to emotional words varying in self-reference (self, other, nobody), the EEG study [30] could demonstrate that, during silent reading, information about the emotionality of a word and its ownership (self vs. other) is first coded separately in the visual processing system before being integrated during subsequent stages of word processing. The subsequent stages identified were indicated by a frontal negativity component and by a late positive potential (LPP), an ERP component that is known to reflect processes of post-semantic integration and memory encoding. Importantly, according to EEG-LORETA, source imaging analyses, facilitated processing of self-related as compared to other-related or unreferenced emotion words in the time-window of the frontal negativity, and the LPP coincided with activity changes in the CMS, including parts in the medial prefrontal cortex and the PACC/precuneus. In terms of the dynamic self-model, the findings strongly support the notion that in healthy subjects, self-emotion interactions may preferentially occur at the Me2 level and are expressed by amplitude modulations of ERPs, which mirror the more elaborate and in depth processing of emotional information. In electrophysiological terms, this stage is initiated temporarily after an earlier processing stage during which ERP components, such as the early posterior negativity (EPN) are elicited, and which mirrors rapid attention capture and early conceptual processing of sensory relevant input [42,43,44]. Modulation of the EPN by emotional content has been repeatedly demonstrated for words, pictures, and faces. For words, Herbert et al. [45] demonstrated that early stimulus-driven changes are likely to be caused by re-entrant processing between the amygdala and the visual processing system. Modulation of early ERPs, such as the EPN, should thus be especially sensitive to processes triggered by aspects of the self for whom a strong evolutionary basis has been theoretically propagated.

Yet, several other ERP studies also suggest early facilitated processing of emotional stimuli in the time window of the EPN to be a robust phenomenon that is not affected by factors such as task demands and self-reflective evaluations [30,42,46,47]. Regarding the latter, the EPN reference effect found in the studies by Herbert et al. [30,32] confirm an early stage of Me1 for visual processing of self-other referential emotional vs neutral words, while Walla et al. used neutral nouns only. However, in all these studies, significant differences in the processing between stimuli with reference (self and other) vs. stimuli with no self-other reference were found, but ERPs did not discriminate between stimuli related to self or other at these primarily pre-reflective processing stages. This particular finding of no distinction between self vs other at early processing stages certainly awaits results from future studies using non-verbal, possibly auditory, or multimodal stimuli to ensure that this effect is not stimulus- or modality specific. So far, the EPN reference effects in Herbert et al.’s study [31,32] in particular, fit nicely with the idea of a primitive ME1 as suggested by Walla, based on his findings. At least for an early stage of processing, many of the studies mentioned in this paper support this notion that self- and other-related symbolic information seems to be commonly coded regardless of whether this information carries emotional meaning or not.

## 5. Introducing Empirical Evidence about Non-Conscious Serial Dynamic Self Aspects

One of the well-known contrasts in cognitive neuroscience is the dissociation between implicit and explicit processing [49,50]. Implicit processing can be understood as processing of information outside awareness while still subserving decision making and guiding behaviour. On the other hand, explicit processing means that one is fully aware of the information being processed. Given that implicit processing in the human brain has been demonstrated numerous times, one wonders whether information about the self is or at least can partially be processed outside awareness. The vast majority of previous studies about the self-utilised experimental paradigms including instructions that forced participants to consciously process self-referential information. Two particular studies though [27,28] used a traditional psychological tool to vary the level of processing across encoding instructions in order to control the depth of semantic verbal processing. This well-known level of processing framework [51] has been proven to successfully involve distinct brain functions in ongoing processing while also manipulating behavioural performance. For instance, conscious semantic encoding increases subsequent recognition performance when compared to only alphabetical encoding, while also being associated with greater brain activity [52].

Obviously, verbal information can be processed outside awareness, a phenomenon that has been shown numerous times in the frame of priming experiments. Prior exposure to a word facilitates its subsequent processing even in the absence of any conscious recollection. Now, the question is what happens if verbal information being processed is self-referential? Walla et al. [27,28] answered this question in their MEG and EEG studies. They used pairs of possessive pronouns and nouns to elicit a sense of ownership or belonging, while varying levels of word information processing across three different experiment sessions. By eliciting ‘self’-referential brain activity on the single word level it becomes much easier to control for level of attention and thus to modify the level of conscious reading. Basically, it means that words are processed in different depths depending on the given instruction. You will only process the letters of a word (alphabetical encoding) to answer the question, whether the first and last letters of the word are in alphabetical order or not. Instead, you need to consciously read the word to answer the question, whether the word’s meaning is living or non-living (semantic encoding). Different depths of word processing result in significantly different subsequent recognition performances. In fact, recognition performance after conscious processing of word meaning is twice as good as after alphabetical encoding, which indicates that alphabetical word encoding indeed minimises conscious processing (if not entirely excludes it) of word meaning.

However, this is not ideal if only pronouns are visually presented. To complete the picture, and in order to make personal engagement elicited by possessive pronouns even more effective, all pronouns were presented together with nouns (word pairs; my garden, his garden, a garden) in the Walla et al. studies. This gives *possession* a more realistic touch. In addition, by using such word pairs, it is possible to direct any task-related attention to nouns and keep it away from pronouns. Applying the above-mentioned encoding instructions (alphabetical encoding and semantic encoding) to nouns only results in widely unconscious pronoun encoding. To finally compare these two unconscious pronoun encoding conditions with a conscious pronoun encoding condition, a third instruction was introduced. Participants were asked to create a short meaningful sentence in their mind, and include both the pronoun and the noun (“my garden is nice”, “his garden is nice”, “a garden is nice”). All three different word-encoding instructions were used in both Walla et al. studies. The crucial finding was that the factor ‘*depth of processing*’ did not significantly interact with the main *pronoun effects* on brain activity. It was thus concluded that the pronoun effects were independent from the level of processing and thus rather unconscious.

It has always been difficult to ensure non-conscious processing in cognitive and affective neuroscience experiments with human subjects. However, the above-mentioned “depth of processing” approach at least demonstrates detectable behavioural effects that reflect different awareness levels. Thus, if not (absolutely) non-conscious, this technique allows us to conclude that, on lower levels of awareness, our brains can still discriminate between self and other. Further investigations are needed to provide stronger support for a non-conscious serial dynamic self.

## 6. Conclusions

A recent review [53] summarised neuroimaging studies of self-referential processing by comparing verbal with non-verbal tasks to elicit self-referential processing. The current short communication focusses only on verbal tasks by highlighting electrophysiological studies demonstrating a temporal and a spatial distinction between two separate neurophysiological phenomena (aspects) related to self-referential processing in the human brain. In response to that, a multiple aspects self-theory (MAST) is postulated including a Me1 and a Me2. This theory is meant to form the basis for future studies that should support the theory and ideally further expand it to better understand how the human brain processes the multiple aspects of self-referential information. In the end, this will help in better understanding the human self in principle.

## Data Availability

Not applicable.
